# Supplementary material for: Decision Tree Ensembles Utilizing Multivariate Splits Are Effective at Investigating Beta Diversity in Medically Relevant 16S Amplicon Sequencing Data
Source: Microbiol Spectr. 2023 Mar 6;11(2):e02065-22. doi: 10.1128/spectrum.02065-22 (PMC10100742; doi:10.1128/spectrum.02065-22)

Supplementary Figure 1: High-level overview of how TreeOrdination produces a projection. (A) Columns in a copy of the training data are randomized. The randomized data is then concatenated to the original data. (B) *N* LANDMark classifiers are trained on *N* unique randomizations of the original data. No information about the original sample labels is used to train these classifiers. (C) Using the training data, the leaf labels for each of the *N* LANDMark classifiers are extracted and concatenated together. The result is a binary matrix where each row is a sample and each column a leaf label. (D) Using this binary matrix, a UMAP/PCA transformer is trained. (E) Using the unseen or testing data, the leaf labels for each of the *N* LANDMark classifiers are extracted and concatenated together. The result is a binary matrix where each row is a sample and each column a leaf label. (F) The trained UMAP/PCA transformer from (D) is then used to transform this matrix.

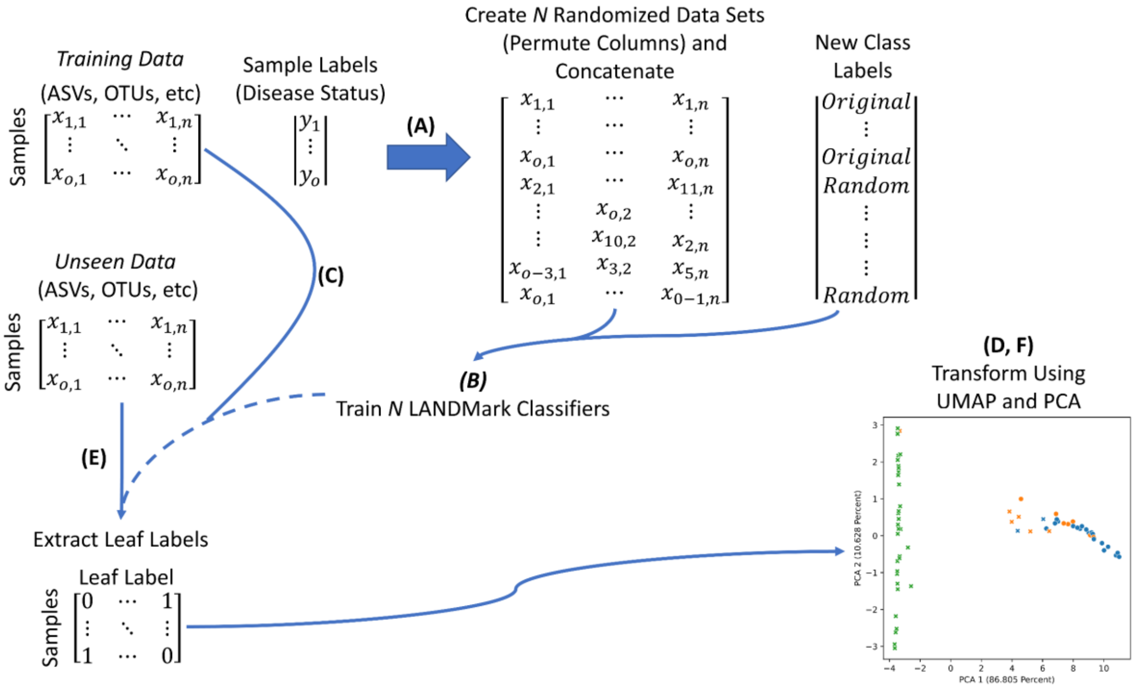

Supplementary Figure 2: Balanced accuracy score results for each test condition in the positive control data. Higher scores indicate a more accurate classifier. The 95% confidence interval, calculated using 2000 bootstraps, is shown for each bar in black. Five-fold stratified cross-validation with five repeats was used to generate this data. The statistical significance of each pairwise comparison was computed using the Wilcoxon test followed by a Benjamini-Hochberg correction. $p \leq 0.0001$ (****), $p \leq 0.001$ (***), $p \leq 0.01$ (**), $p \leq 0.05$ (*), $p > 0.05$ (ns).

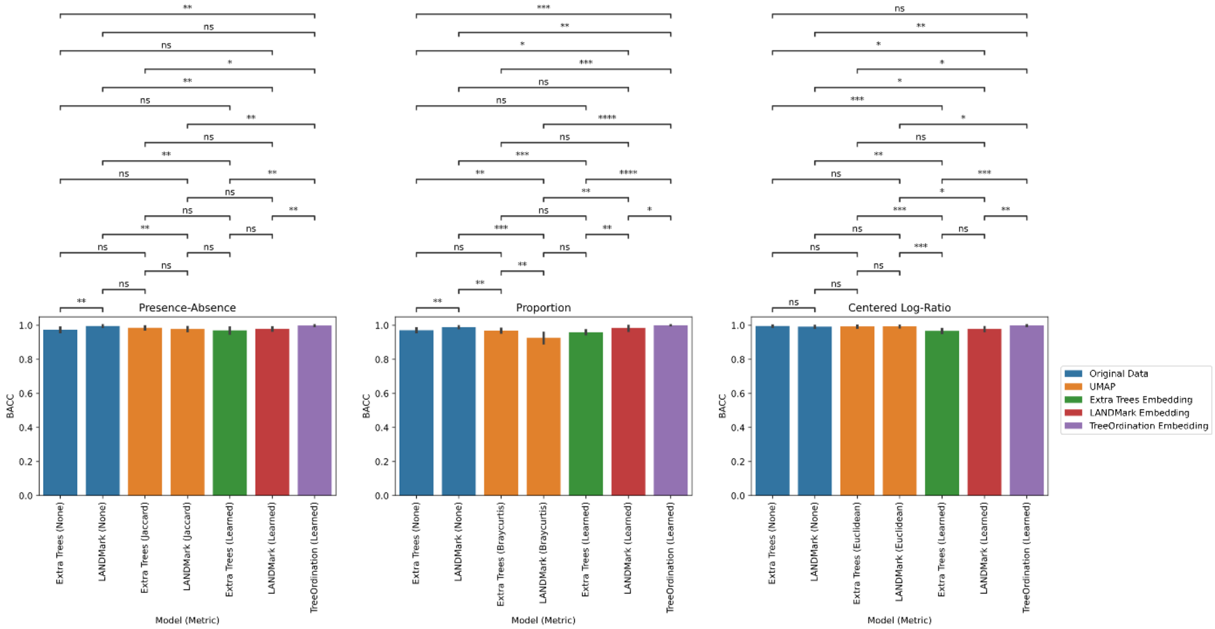

Supplementary Figure 3: Balanced accuracy score results for each test condition in the Crohn's Disease data. Higher scores indicate a more accurate classifier. The 95% confidence interval, calculated using 2000 bootstraps, is shown for each bar in black. Five-fold stratified cross-validation with five repeats was used to generate this data. The statistical significance of each pairwise comparison was computed using the Wilcoxon test followed by a Benjamini-Hochberg correction. $p \leq 0.0001$ (****), $p \leq 0.001$ (***), $p \leq 0.01$ (**), $p \leq 0.05$ (*), $p > 0.05$ (ns).

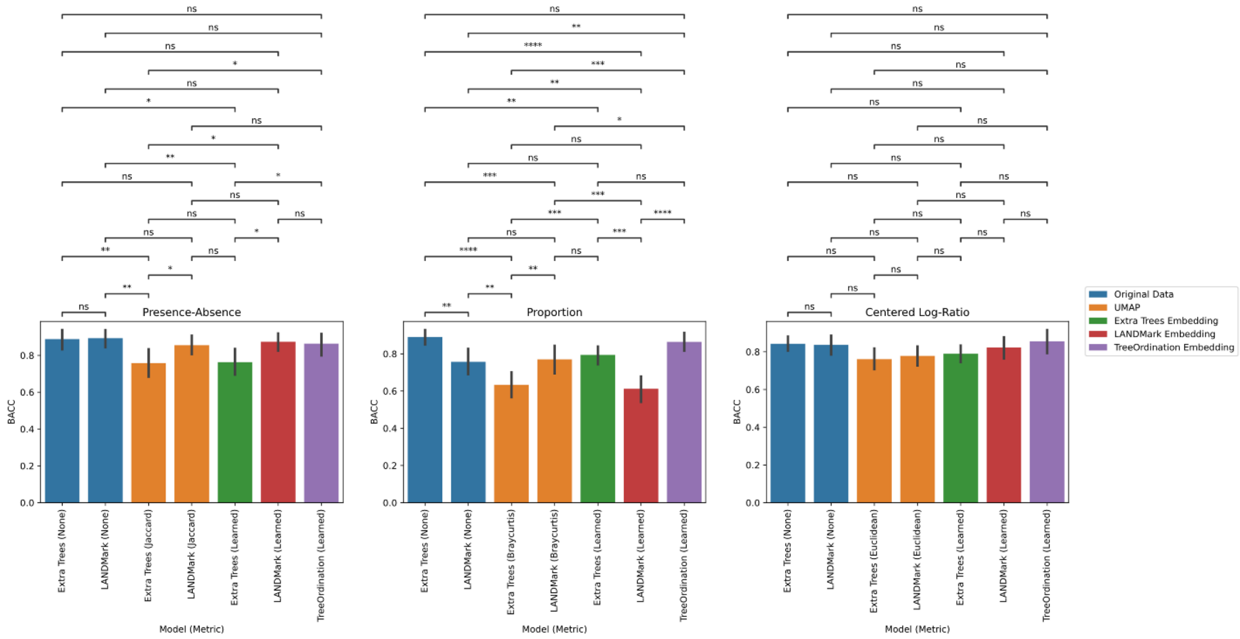

Supplement: Supplemental file 1 — Fig. S1 to S3. Download spectrum.02065-22-s0001.pdf, PDF file, 0.4 MB [file spectrum.02065-22-s0001.pdf]
